# Acute and sub-acute toxicity study of aqueous and methanol root extract of *Tetracera alnifolia* in male albino rats

**DOI:** 10.1016/j.toxrep.2024.101786

**Published:** 2024-10-22

**Authors:** Irene Ebosereme Ainyanbhor, Iyere Osolase Onoagbe, Great Iruoghene Edo, Emad Yousif, Patrick Othuke Akpoghelie, Joseph Oghenewogaga Owheruo, Endurance Fegor Isoje, Ufuoma Augustina Igbuku, Arthur Efeoghene Athan Essaghah, Huzaifa Umar

**Affiliations:** a Department of Biochemistry, Faculty of Science, Delta State University of Science and Technology, Ozoro, Delta State, Nigeria; b Department of Biochemistry, Faculty of Life Science, University of Benin, Benin, Nigeria; c Department of Chemistry, Faculty of Science, Delta State University of Science and Technology, Ozoro, Nigeria; d Department of Chemistry, College of Sciences, Al-Nahrain University, Baghdad, Iraq; e Department of Food Science and Technology, Faculty of Science, Delta State University of Science and Technology, Ozoro, Delta State, Nigeria; f Department of Science Laboratory Technology (Biochemistry Option), Faculty of Science, Delta State University of Science and Technology, Ozoro, Nigeria; g Department of Urban and Regional Planning, Faculty of Environmental Sciences, Delta State University of Science and Technology, Ozoro, Delta State, Nigeria; h Operational Research Centre in Healthcare, Near East University, Nicosia, Cyprus

**Keywords:** Toxicity, *Tetracera alnifolia*, Superoxide dismutase, Antioxidant, phytochemicals

## Abstract

The aim of this study was to assess the acute and sub-acute toxicity of aqueous and methanol extracts of the root of *Tetracera alnifolia* as well as the effects on some biochemical parameters in albino rats as many plants used in traditional medicine lack scientific and clinical evidence to support a better understanding of their safety and efficacy. Phytochemical screening and proximate analysis of the pulverised root of *Tetracera alnifolia* was carried out using previously reported protocol. Sub-acute toxicity study of each extract was done for 28 days followed by organs function tests and histopathology studies of the liver, kidney and heart. Evaluation of lipid profile and oxidative stress marker to ascertain the effect of each extract on lipid peroxidation and their antioxidant property was done after administration of 200 mg/Kg body weight of each extract for a period of thirty-five days. Acute toxicity study of each extract gave oral LD_50_ (rat) of greater than 5000 mg/kg body weight with no signs of toxicity. Sub-acute toxicity study showed both extracts were non-toxic to the liver, kidney, heart and blood at doses between 200 and 3000 mg/Kg body weight assessed by the respective organ function tests, hematological parameters, and histopathology study. However, higher doses seem toxic to the liver particularly at 5000 mg/kg B. W due to increase in plasma AST, ALT and ALP activities accompanied with reduced protein and albumin concentrations. Effects of each extracts at 200 mg/Kg body weight on some biochemical parameters revealed no significant difference in lipid profile parameters and no lipid peroxidation. Each extract may possess antioxidant property due to increase in catalase activity. The result from this research may help validate the safety of the oral use of this plant in traditional medicine.

## Highlights

• The oral LD_50_ for aqueous and methanol root extracts of *Tetracera alnifolia* in albino rats was greater than 5000 mg/kg body weight.
• The experimental animals seem safe from the risk of developing cardiovascular disease resulting from derailment in lipid metabolism.
• This may imply that the aqueous extract of the root of *Tetracera alnifolia* has a greater potential of lowering the intrinsic factors that cause myocardial infarction

## Graphical Abstract

**Figure:**
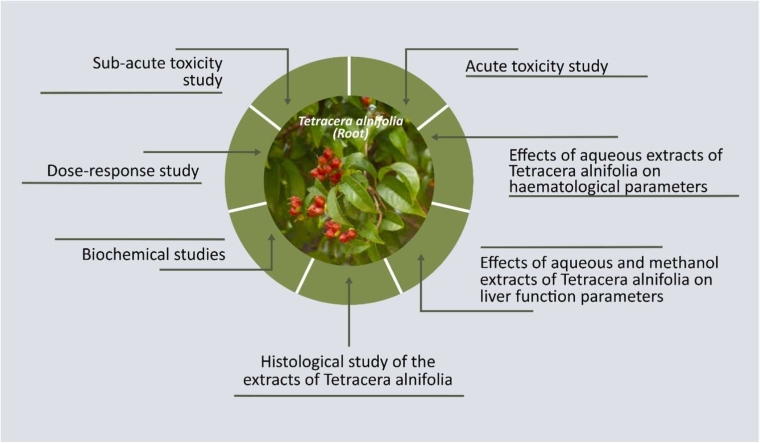

## 1 Introduction

Although plants have been used for generations as source of medicines, scientists and the general public now understand the value of plants as a source of novel or complementary medicinal products [1]. Numerous studies conducted over-time have demonstrated the potential health benefits of plant-based medicinal products [2]. A significant number of the world's population receives their primary medical care basically from plant-based traditional medicine system, which continues to play a significant role in healthcare [3]. Medicinal plants are the major bio-resource of drugs of traditional medicine, modern medicines, nutraceuticals, food supplements, folk medicines, pharmaceutical intermediates and chemical entities for synthetic drugs [4].

A medicinal plant is any plant in which one or more of its organs such as root, stem and leaf contains substances that can be used for therapeutic purpose or are precursors for the synthesis of useful drugs [5]. It can also be referred to as any plant which provides health-promoting characteristics, temporary relief to symptomatic problem or has curative properties [6]. Medicinal plants are of great importance to the health of individuals and communities [7]. The medicinal value of these plants lies in some chemical substances that produce definite physiological action on the human body [8]. The presence of bioactive components in plants, such as alkaloids, phenols, flavonoids, tannins, glycosides, and essential oils, is what determines their medicinal qualities [9]. This makes it necessary to keep screening medicinal plants in order to find new active components as well as the scientific basis for their safety and therapeutic application [10]. Plant kingdom holds many species of plants containing bioactive compounds of medicinal value [11]. From time immemorial till date, plant materials have been used in the treatment of many diseases and infections [12].

In developing countries of the world like Nigeria, most people still depend on herbal medical care that uses mainly medicinal plant for treatment [13]. The use of medicinal plants especially in traditional medicine is currently well-acknowledged and established as a viable option in Nigeria in the treatment and management of many diseases [14].

One of the medicinal plants used in traditional medicine in Nigeria is *Tetracera alnifolia* popularly as known as *Opon* in Yoruba language [15]. *Tetracera alnifolia* is a climbing shrub forest plant, occasionally epiphytic, with white flowers [16]. It is used traditionally for the treatment of arthritis, rheumatism, anaemia and diabetes mellitus [17]. In Guinea, it is used to treat sexually transmitted infections, skin disease, and malaria [18]. Methanol and chloroform extracts of *Tetracera alnifolia* has been reported to have *invitro* antimicrobial property on *Trypanosoma*. *Leishmania* and SAR-CoV-2 virus while the aqueous extract on *Staphylococcus aureus* [19]. Methanol extract of *Tetracera alnifolia* has been shown to exhibit antibacterial property on *Staphylococcus aureus*, *Streptococcus pneumoniae*, *Pseudomonas aeruginosa* and *Proteus mirabilis* as well as antifungal on the dermatophytes *Trichophyton rubrum* and *Microsporum canis* [20]*.*

Although plants are still the primary source of drugs in traditional and alternative system of medicine because of the decreasing efficacy [21], non-affordability, and increasing contra-indications of the use of synthetic drugs, their usage of in the treatment and management of various diseases and ailments lack scientific basis [22]. The lack of clinical and scientific evidence to support a better understanding of the safety and effectiveness of the medications is the primary draw back to the use of traditional herbal preparations [23]. This is because there hasn't been toxicity study and adverse drug reaction study done on most herbal medicine used in traditional medicine since plant products are mistakenly believed to be natural and thus safe [24]. Some extracts from plants may contain harmful toxins that are carcinogenic or cytotoxic to cells [25]. It is therefore important to study medicinal plants in details in order to establish their safety through toxicological and biochemical assessment [26]. The aim of this research study was to investigate the effect of methanol and aqueous extract of the root *Tetracera alnifolia* on some biochemical parameter and also their toxicological studies in male albino rats.

## 2 Materials and methods

### 2.1 Materials

#### 2.1.1 Chemicals

Disodium hydrogen phosphate, sodium dihydrogen phosphate, trichloroacetic acid, thiobarbituric acid and sodium hydroxide were supplied by Kermel, China. Hydrochloric acid, hydrogen peroxide, potassium permanganate, epinephrine, sodium carbonate, pyrogallol were supplied by Sigma-Aldrich, USA, tetraoxosulphate (iv) acid (Fisons, UK), Ellman’s reagent (Thermofisher scientific, USA), ethylene diamineteraacetic acid (EDTA) (BDH, India), sodium bicarbonate (Merck, Germany), sodium chloride (Lobachem, India) and methanol (JHD, China)

#### 2.1.2 Reagents

The following reagents (kits) were supplied by Randox Laboratories Limited, County Antrim, United Kingdom: cholesterol, triglyceride, HDL-cholesterol, aspartate aminotransferase, alanine aminotransferase, L-gamma glutamyltransferase, bilirubin, total protein, albumin, urea, creatinine and lactate dehydrogenase. Sodium, potassium, chloride, bicarbonate and alkaline phosphatase reagents (kits) were supplied by Teco Diagnostics, Anaheim, USA while creatine kinase was supplied by Agappe Diagnostics, Switzerland.

#### 2.1.3 Animals

Male Wistar albino rats between the weight of 140 g to 160 g obtained from the animal house, Department of Pharmacology, Faculty of Pharmacy, University of Benin, Benin City, Nigeria were used for this study. The rats were kept in wooden cages in the departmental animal house and were allowed to acclimatize for two weeks before commencement of the experiment. They were given free access to water and food all through the period of study.

Experiments conducted in this study followed the European convention for the protection of vertebrate animals used for experimental and other specific purposes. The Animal Ethics Committee, University of Benin, Benin City, Nigeria (023/0345) approval was obtained before commencement of the procedures.

#### 2.1.4 Plant material

Fresh leaves and roots of the plant were obtained from Oyingbo market in Lagos State. The leaves were identified in the Department of Plant biology and Biotechnology, University of Benin. Herbarium specimen with voucher number UBH_T258_ was deposited at the Department of Plant Biology and Biotechnology herbarium. The roots were properly washed with water, cut and dried at room temperature for four weeks in the departmental laboratory. The roots were pulverized and 500 grammes of the pulverized root were soaked in 2500 mL of water and methanol respectively for 72 hours with intermittent stirring [27]. The mixtures were filtered using a muslin cloth. Both extracts were freeze dried using a lyophylizer and kept in a refrigerator until when needed.

The percentage yield was calculated as: % yield of extract =$\frac{\mathrm{Weight}\;\mathrm{of}\;\mathrm{extract}}{\mathrm{Weight}\;\mathrm{of}\;\mathrm{pulverised}\;\mathrm{root}\;\mathrm{used}}\times \mathrm{100}$

The percentage yield for aqueous and methanol extract was 5.6 % and 7.1 % respectively.

### 2.2 METHODS

#### 2.2.1 Proximate Analysis

The estimation of the nutritional composition (carbohydrate, crude lipid, protein, fibre, moisture and ash content) in pulverized root of *Tetracera alnifolia* was carried out according to the methods of [28] [29].

#### 2.2.2 Phytochemical screening

The extracts of the root of *Tetracera alnifolia* were screened for the presence of alkaloids, saponins, anthraquinones, steroids, tannins, flavonoids, reducing sugars, terpernoids and cardiac glycosides according to standard methods [2], [1], [30].

#### 2.2.3 Acute toxicity study

This was done for each extract using Lorke method [1]. This method involves two stages namely: stage 1 and stage 2.

**Phase 1:** Eighteen albino rats weighing between140–160 grammes were obtained and divided into aqueous extract and methanol extract groups. Aqueous extract and Methanol extract groups were subdivided into three sub-groups (three rats per group). The sub-groups of the aqueous and methanol extract group were administered 10, 100 and 1000 mg/Kg body weight of the extract.

The rats were closely observed for physical signs of toxicity such as changes in breathing, numbness, loss of appetite, salivation, convulsion, weakness or aggressiveness, water intake, sluggishness, weight loss, tremor and death for a period of 72 hours.

**Phase 2:** Eight albino rats weighing between 140 and 160 grammes were obtained and divided into aqueous and methanol extract groups. Each of the groups was further divided into four sub-groups (one rats/group). The various sub-groups of the aqueous and methanol extract group were administered 1500, 2500 and 3500 and 5000 mg/Kg body weight of their respective extract.

The rats were closely observed for signs of toxicity such as changes in breathing, numbness, loss of appetite, salivation, convulsion, weakness or aggressiveness, water intake, sluggishness, weight loss, tremor and death for 72 hours.

#### 2.2.4 Sub-acute toxicity study

This study was done for each extract according to [1]. Ninety rats weighing between 140 and 160 grammes were obtained and divided into aqueous and methanol extract group. Each group was further divided into seven sub-groups (six rats per group). The control group had six rats. The sub-groups of the aqueous extract group were administered 200, 500, 1000, 2000, 3000, 4000 and 5000 mg/Kg body weight. Also, the sub-groups of the methanol group were administered 200, 500 1000, 2000, 3000, 4000 and 5000 mg/Kg body weight**.**

Weekly checks were made on the changes in body weight during the experimented period. Similar to the acute toxicity study, the animals were watched for signs of toxicity including changes in food intake and death. After the period of administration of extracts (28 days), the animals in each sub-group and control group were anaesthetized on the 29th day by intraperitoneal injection of 150 mg/Kg pentobarbital. Blood was collected from each rat's lower aorta using a hypodermic needle and syringe. The blood samples were collected in properly labeled blood collection bottles containing EDTA and lithium heparin as anticoagulants. The heart, kidney and liver were excised and kept in containers containing 10 % neutral buffered formalin for histopathology study. Blood samples containing lithium heparin were centrifuged at 4000 rpm for ten minutes. The plasma was then separated in a plain container and used for the various assays.

**Kidney function test:** The following assays were done to assess kidney function of the animals: sodium, potassium, chloride, bicarbonate, creatinine and urea.

**Liver function test:** The assays to assess liver function in the rats included: alanine aminotransferase (ALT) activity, L-y-glutamytransferase activity, alkaline phosphate activity (ALP), bilirubin (total and direct) concentration, total protein and albumin concentration.

**Heart function test**: The assays to assess heart function in the rats included: lactate dehydrogenase (LD), aspartate aminotransferase activity (AST) and creatine kinase (CK) activity.

**Haematological test:** The EDTA blood samples were used for haematology test using a PCE-210E Version 5.42 N haematology analyzer. The following haematological parameters were performed on the blood sample: haematocruit, haemoglobin concentration, mean cell volume, mean cell haemoglobin concentration, total and differential white blood cell count and platelet count.

**Histological study:** The excised organs (liver, kidney and heart) were kept in a universal container containing 10 % neutral buffered formalin, processed and stained for histopathology study following standard procedure [31].

#### 2.2.5 Dose-response study

Thirty-six albino rats weighing between 140 and 160 grammes were obtained and divided into aqueous, methanol and control groups. The methanol and aqueous groups were further divided into three groups (six rats per group). The control group was made up of six rats. They were acclimatized to their environment for 14 days and their blood sugar was checked using a glucometer on the 15th day after an overnight fasting for 12 hours. The sub-groups of the aqueous extract group were administered 200, 500 and 1000 mg/Kg body weight. Also, the sub-groups of the methanol group were administered 200, 500 and 1000 mg/Kg body weight**.** The blood sugar of each rat in each group was check consecutively every three days after an overnight fasting for 12 hours before feeding in the morning. The blood glucose of each animal in the various groups was monitored during the period of study for significant reduction. The values obtained were used to plot a dose response graph for each extract.

#### 2.2.6 Biochemical profile study

Twenty- four rats weighing 140–160 grammes were obtained. The animals were divided into three groups namely: control group, aqueous extract group, and methanol extract groups. Each group was made up of six rats. The methanol and aqueous group were given 200 mg/kg body weight (the result of the dose response study) once daily for thirty-five days while the control group was given water.

At the end of the period of study, the rats were fasted overnight and were sacrificed. Each animal in each group was anaesthetized with 150 mg/Kg pentobarbital intraperitoneal injection. Hypodermic needle and syringes were used to obtain blood from lower aorta of each rats. The blood samples were collected in properly labeled blood collection bottles containing Lithium heparin as anticoagulants. They were centrifuged at 4000 rpm for 10 minutes; the plasma was separated in a plain container and used for the following assays: cholesterol, triglycerides, HDL-cholesterol, superoxide dismutase (SOD), catalase (CAT), glutathione peroxidase, reduced glutathione and malondialdehyde.

#### 2.2.7 Assay methodology

Plasma ALT activity was determine by the method of [32], AST [33], L-Gamma Glutamyltransferase (GGT) activity [34], ALP [35], bilirubin concentration (Total and Direct) [36], total protein concentration [37], and albumin concentration [38]. Plasma Urea concentration was determined according to the method of [39], creatinine concentration [40], sodium ion concentration [41], potassium ion concentration [42], chloride ion concentration [43], and bicarbonate ion concentration [44]. Plasma LD activity was determined by the method of [45] and CK activity by the method of [46]. Plasma triglyceride concentration was determined according to the method of [47], cholesterol concentration [48], HDL-cholesterol concentration [49]. CAT activity was determined by the method of [50], superoxide dismutase activity [51], glutathione peroxidase activity [1], reduced glutathione [52], and malondialdehyde was determined using the thiobarbituric acid method [53].

#### 2.2.8 Statistical analysis

All results were expressed as mean ± standard deviation (S. D) of six rats and were analyzed using analysis of variance (ANOVA) using one-way test. Less significance difference (LSD) was used in a post hoc test multiple comparison to ascertain the significance of the mean difference between each treatment group and the control group. Test significance was considered at p < 0.05. SPSS version 17.0 was used for the analysis.

## 3 Results

### 3.1 Proximate analysis

Proximate analysis of the pulverized root of *Tetracera alnifolia* revealed that carbohydrate was the highest nutrient while moisture content was the least (Table 1).

**Table 1:** Nutritional composition of the pulverized root of *Tetracera alnifolia*.

Nutrients	Percentage composition
Moisture content	5.05±0.15
Ash content	10.00±0.17
Crude Fat	10.13±0.13
Crude Fibre	15.32±0.01
Protein	5.25±0.00
Carbohydrates	54.25±0.08

### 3.2 Phytochemical screening

Phytochemical screening of the methanol and aqueous extracts of root of *Tetracera alnifolia* revealed the presence of alkaloid, flavonoid, saponins, tannins, phenols, phlobatannins, cardiac glycosides, anthraquinone, and steroid in both extracts (Table 2).

**Table 2:** Phytochemicals present in aqueous and methanol root extract of *Tetracera alnifolia*.

Phytochemicals	Aqueous Extract	Methanol Extract
Alkaloids	+	+
Saponins	+	+
Tannins	+	+
Phenols	+	+
Cardiac glycosides	+	+
Anthraquinone	+	+
Steroid	+	+
Flavonoids	+	+

+ Present, - absent

### 3.3 Acute toxicity study

The acute toxicity study of aqueous and methanol extract of root of *Tetracera alnifolia* in albino rats is presented in Table 3**.** From the acute toxicity study, physical observation of individual animals in each group showed that there were no signs of toxicity such as changes in breathing, numbness, loss of appetite, salivation, convulsion, weakness, aggressiveness, reduced water intake, sluggishness, weight loss, tremor and death. Using Lorke method, since there was no death during the period of study for both extracts, the oral lethal dose (LD_50_) for each extract in Wistar albino rat was greater than 5000 mg/kg body weight.

**Table 3:** Acute toxicity study of aqueous and methanol extracts of the root of *Tetracera alnifolia.*

Dose (mg/kg body weight) Phase 1	Aqueous extract Observation (Mortality)	Methanol extract Observation (Mortality)
10	0/3	0/3
100	0/3	0/3
100	0/3	0/3
Phase 2		
1500	0/3	0/3
2500	0/3	0/3
3500	0/3	0/3
5000	0/3	0/3

### 3.4 Sub-acute toxicity study

During the period of sub-acute toxicity study, physical observation of individual animals in each group showed that there were no signs of toxicity such as changes in breathing, numbness, loss of appetite, salivation, convulsion, weakness, aggressiveness, reduced water intake, sluggishness, weight loss, tremor and death

#### 3.4.1 Effect of aqueous and methanol extracts of *Tetracera alnifolia* on heart function parameters

Table 4a showed there was no significant difference in AST activity in rats at doses 200–3000 mg/Kg body weight aqueous extract except at doses 4000 and 5000 mg/kg body weight with the enzyme activity at 37.83 U/L and 38.48 U/L respectively and were significantly higher than the control (27.17 U/L). There was no discernible difference in LD activity across all the groups when compared with the control. Creatine kinase activity was significantly reduced at doses 200–4000 mg/Kg body weight except at dose 5000 mg/kg body weight where it was not significantly different from the control.

**Table 4a:** Effect of aqueous extract of *Tetracera alnifolia* on heart function parameters.

DOSE (mg/kg bw)	AST(U/L)	LDH(U/L)	CK(U/L)
CONTROL	27.17±1.80^a^	78.41±7.90^a^	18.57±2.06^a^
200	25.53±1.90^a^	71.54±6.00^a^	5.15±1.37 ^y^
500	26.26±1.91^a^	89.77±4.58^a^	5.16±1.03 ^y^
1000	28.13±0.41^a^	82.54±19.36^a^	5.50.±1.03 ^y^
2000	30.30±1.82^a^	71.31±11.10^a^	6.19±2.06 ^y^
3000	31.49±2.48^a^	69.50±7.12^a^	7.22±1.22 ^y^
4000	37.83±3.97^x^	96.98±7.05^a^	11.34±3.52 ^y^
5000	38.48.±1.10^x^	85.60±5.94^a^	20.64±3.36^a^

Table 4b showed that at doses 4000 and 5000 mg/kg body weight, the plasma aspartate aminotransferase activity of the rats administered methanol extract was 38.57 U/L and 38.26 U/L respectively and were considerably higher than the control with the enzyme activity at 27.17 U/L. The plasma creatine kinase and lactate dehydrogenase activities were not significantly different from the control for the various doses.

**Table 4b:** Effect of methanol extract of *Tetracera alnifolia* on heart function parameters.

DOSE (mg/kg bw)	AST(U/L)	LDH(U/L)	CK(U/L)
CONTROL	27.17±1.80^a^	78.41±7.90^a^	18.57±2.06^a^
200	30.09±3.35^a^	96.98±9.60^a^	16.51±4.46^a^
500	30.12±1.77^a^	76.35±7.98^a^	19.6±2.60^a^
1000	31.11±1.53^a^	53.64±9.97^a^	15.48±2.60^a^
2000	32.09±2.54^a^	68.79±6.88^a^	12.36±2.40^a^
3000	31.06±2.03^a^	52.62±6.61^a^	14.44±2.66^a^
4000	38.57±2.10^x^	78.44±13.69^a^	13.41±2.60^a^
5000	38.26±3.65^x^	74.28±0.00^a^	15.01±1.38^a^

Values are enzyme activities in Units/L and are expressed as Mean ± S.D. Values with the same superscript as Control are not significantly different. Values with superscript x are significantly increased when compared to control while values with superscript y are significantly decreased when compared to control.

#### 3.4.2 Effect of aqueous and methanol extracts of *Tetracera alnifolia* on Kidney function parameters

Table 5a, Table 5b showed plasma concentrations of urea, creatinine, sodium, chloride, and bicarbonate were significantly different from the control for the different doses of aqueous and methanol extracts. While there was no significant difference in potassium ion concentrations at doses 200–5000 mg/Kg body weight methanol extract, potassium ion concentration at dose 5000 mg/kg body weight aqueous extract was 5.40 mEq/L and was significantly higher that the control which was 3.84 mEq/L.

**Table 5a:** Effect of aqueous extract of *Tetracera alnifolia* on kidney function parameters.

Dose(mg/kg bw)	Sodium (mEq/L)	Chloride (mEq/L)	Potassium (mEq/L)	Bicarbonate (mmol/L)	Urea (mg/dL)	Creatinine (mg/dl)
CONTROL	139.45±2.04^a^	103.57±1.58^a^	3.84 ±0.30^a^	25.60 ±1.17^a^	18.20 ±1.12^a^	1.01±0.04^a^
200	137.21±2.10^a^	104.64±3.28^a^	4.51 ±0.86^a^	25.79 ±1.30^a^	22.92 ±0.51^a^	0.61±0.09^a^
500	135.9±3.68^a^	98.06±1.30^a^	4.00 ±0.07^a^	25.43 ±1.12^a^	16.65 ±2.04^a^	0.98±0.05^a^
1000	137.35±3.66^a^	97.58±2.82^a^	4.72 ±0.28^a^	26.33 ±1.95^a^	14.76 ±2.44^a^	1.00±0.03^a^
2000	138.04±3.76^a^	105.83±1.20^a^	4.72 ±0.36^a^	25.83 ±1.09^a^	16.96 ±0.51^a^	1.09±0.10^a^
3000	133.88±1.82^a^	101.65±1.77^a^	4.64 ±0.36^a^	25.85 ±1.09^a^	20.12 ±1.14^a^	1.27±0.07^a^
4000	136.80±2.31^a^	101.57±1.57^a^	4.55 ±0.54^a^	27.84 ±0.97^a^	15.73 ±1.80^a^	1.16±0.10^a^
5000	135.50±1.81^a^	103.81±2.39^a^	5.40 ±0.22^x^	26.49 ±1.60^a^	17.88 ±2.66^a^	1.06±0.04^a^

**Table 5b:** Effect of methanol extract of *Tetraceraalnifolia* on Kidney function parameters.

**Dose mg/Kg bw**	**Sodium (mEq/L)**	**Chloride (mEq/L)**	**Potassium (mEq/L)**	**Bicarbonate (mmol/L)**	**Urea (mg/dL)**	**Creatinine (mg/dl)**
CONTROL	139.45 ±2.04^a^	103.57 ±1.58^a^	3.84±0.30^a^	25.60±1.17^a^	18.20±1.12^a^	1.01±0.04^a^
200	136.75 ±0.66^a^	98.22 ±0.40^a^	3.69±0.09^a^	27.25±0.32^a^	13.35±0.58^a^	0.99±0.08^a^
500	133.53 ±2.88^a^	99.51 ±3.53^a^	4.34±0.42^a^	27.85±0.55^a^	13.75±1.74^a^	1.05±0.04^a^
1000	138.78 ±0.81^a^	98.71 ±1.05^a^	4.21±0.39^a^	27.14±1.10^a^	14.03±1.05^a^	0.95±0.26^a^
2000	137.78 ±2.35^a^	100.58 ±0.15^a^	3.91±0.52^a^	28.52±1.83^a^	16.27±2.17^a^	1.00±0.03^a^
3000	135.21 ±2.14^a^	98.24 ±2.23^a^	3.41±0.35^a^	26.90±0.55^a^	17.50±3.21^a^	1.18±0.12^a^
4000	134.51 ±3.23^a^	98.88 ±3.53^a^	3.91±0.70^a^	26.76±0.67^a^	19.88±2.40^a^	1.34±0.20^a^
5000	138.50 ±0.36^a^	102.84 ±0.15^a^	5.04±0.29^a^	26.51±0.11^a^	20.50±1.10^a^	1.07±0.05^a^

Values are concentrations of some kidney function parameters and are expressed as Mean ± S.D (Standard Deviation). Values with the same superscript as Control are not significant different. Values with superscript x are significantly increased when compared to control while values with superscript y are significantly decreased when compared to control.

#### 3.4.3 Effect of aqueous and methanol extracts of *Tetracera alnifolia* on liver function parameters

Table 6a, Table 6b showed the plasma protein and albumin concentrations of rats administered the graded doses of aqueous and methanol extract of *Tetracera aalnifolia* were not significantly different from the control except at dose 5000 mg/kg body weight. The protein and albumin concentrations at 5000 mg/Kg body weight aqueous extract were 5.77 g/dL and 3.98 g/dL, methanol extract was 5.59 g/dL and 3.21 g/dL while the control was 6.74 g/dl and 4.74 g/dL respectively. ALT and alkaline phosphatase activities of aqueous extract were 26.76 U/L and 57.43 U/L respectively at dose 5000 mg/kg body weight and were significantly higher than the control at 12.44 U/L and 42.39 U/L respectively. Also, the ALT and ALP activities for the methanol extract were 21.59 U/L and 54.81 U/L and were also significantly increased. Plasma γ-glutamyltransferase activity for each extract did not differ significantly from the control across the various doses. The table also indicated there was no significant difference in plasma concentrations of total and direct bilirubin with the control for each extract.

**Table 6a:** Effect of aqueous extract *Tetracera alnifolia* on liver function parameters.

DOSE (mg/kg bw)	PROTEIN (g/dL)	ALBUMIN (g/dL)	ALT (U/L)	ALP (U/L)	GGT (U/L)	T.BIL (mg/dl)	D.BIL (mg/dl)
CONTROL	6.74±0.25^a^	4.74±0.22^a^	12.44±0.51^a^	42.39±3.35^a^	3.19±0.55^a^	0.20±0.01^a^	0.13±0.02^a^
200	6.20±0.05^a^	3.67±0.65^a^	18.54±0.94^a^	44.70±3.20^a^	1.93±0.77^a^	0.15±0.02^a^	0.06±0.01^a^
500	6.33±0.31^a^	4.24±0.16^a^	19.69±3.84^a^	48.50±3.79^a^	2.32±0.62^a^	0.24±0.07^a^	0.13±0.02^a^
1000	6.27±0.45^a^	4.27±0.30^a^	20.53±1.65^a^	46.07±3.68^a^	3.48±1.06^a^	0.35±0.19^a^	0.11±0.02^a^
2000	6.01±0.33^a^	4.87±0.33^a^	18.93±2.86^a^	47.70±4.14^a^	2.61±0.29^a^	0.34±0.11^a^	0.16±0.04^a^
3000	6.74±0.37^a^	4.43±0.36^a^	18.05±5.19^a^	45.39±0.99^a^	2.61±0.73^a^	0.31±0.20^a^	0.18±0.07^a^
4000	6.83±0.37^a^	4.02±0.44^a^	16.57±4.88^a^	49.89±3.58^a^	3.19±0.55^a^	0.32±0.10^a^	0.16±0.02^a^
5000	5.77±0.19 ^y^	3.08±0.58 ^y^	26.76±1.33^x^	57.43±1.80^x^	3.76±0.55^a^	0.19±0.03^a^	0.11±0.02^a^

**Table 6b:** Effect of methanol extracts of *Tetracera alnifolia* on liver function parameters.

Dose mg/kg bw	Protein g/dL	Alumin g/dL	ALT U/L	ALP U/L	GGT U/L	T.Bil mg/dL	D.Bil mg/dL
CONTROL	6.74±0.25^a^	4.74±0.22^a^	12.44±0.51^a^	42.39±3.35^a^	3.19±0.55^a^	0.20±0.01^a^	0.13±0.02^a^
200	6.65±0.22^a^	4.07±0.44^a^	19.39±1.90^a^	50.54±4.56^a^	3.19±0.98^a^	0.36±0.12^a^	0.14±0.03^a^
500	6.31±0.25^a^	3.57±0.45^a^	16.00±0.98^a^	40.16±2.13^a^	2.61±0.29^a^	0.27±0.02^a^	0.14±0.03^a^
1000	6.84±0.34^a^	4.48±0.28^a^	19.63±2.05^a^	38.71±5.23^a^	3.48±0.47^a^	0.15±0.03^a^	0.07±0.00^a^
2000	6.21±0.16^a^	3.73±0.69^a^	10.83±0.69^a^	39.29±2.59^a^	4.18±0.71^a^	0.15±0.02^a^	0.07±0.00^a^
3000	6.68±0.29^a^	3.94±0.39^a^	11.66±3.23^a^	39.53±3.72^a^	2.32±0.67^a^	0.12±0.01^a^	0.09±0.02^a^
4000	6.62±0.12^a^	4.10±0.24^a^	15.70±0.77^a^	50.43±3.72^a^	1.45±0.29^a^	0.18±0.05^a^	0.13±0.03^a^
5000	5.59±0.28 ^y^	3.21±0.72 ^y^	21.59±1.75^x^	54.81±5.26^x^	3.09±0.38^a^	0.15±0.04^a^	0.12±0.05^a^

The values represent liver function parameter and are presented as Mean ± S D. Values that have the same superscript as the control do not differ significantly. In comparison to the control, values with superscript x show a significant increase, while values with superscript y show a significant decrease.

#### 3.4.4 Effect of aqueous and methanol extracts of *Tetracera alnifolia* on haematological parameters

Table 7a, Table 7b showed that there was no significant difference in haemoglobin concentration, mean cell volume (MCV), mean corpuscular hemoglobin (MCH) and mean corpuscular haemoglobin concentration (MCHC) for the graded doses of each extract. However, there was a significant increase in haematocruit value at 5000 mg/kg body weight for methanol extract (45.20 %) compared with control (40.68 %).

**Table 7a:** Effect of aqueous extract *Tetracera alnifolia* on red blood cells parameters.

DOSE mg/kg bw	HGT g/dl	HCT %	MCV Fl	MCH Pg	MCHC g/dL
CONTROL	13.73±0.34^a^	40.68±1.14^a^	54.73±0.60^a^	18.45±0.13^a^	33.68±0.26^a^
200	12.17±1.20^a^	37.90±4.40^a^	51.07±1.63^a^	18.70±0.50^a^	32.17±0.63^a^
500	13.13±0.33^a^	40.60±2.48^a^	53.60±0.40^a^	17.40±0.31^a^	31.83±0.11^a^
1000	14.13±0.45^a^	44.30±2.88^a^	55.63±1.42^a^	17.73±0.35^a^	31.83±0.27^a^
2000	14.35±0.56^a^	44.63±3.58^a^	56.15±0.09^a^	18.68±0.36^a^	28.23±3.88^a^
3000	14.25±0.18^a^	44.23±3.64^a^	55.87±1.64^a^	17.95±0.41^a^	32.15±0.43^a^
4000	13.68±0.57^a^	42.65±2.56^a^	55.50±0.88^a^	17.78±0.35^a^	32.00±0.48^a^
5000	13.20±0.21^a^	40.48±1.49^a^	58.90±0.47^a^	16.88±0.14^a^	32.55±0.27^a^

**Table 7b:** Effect of methanol extract of *Tetracera alnifolia* on red blood cells parameters.

DOSE mg/kg bw	HGT g/dl	HCT %	MCV Fl	MCH Pg	MCHC g/dl
CONTROL	13.73±0.34^a^	40.68 ±1.14^a^	54.73±0.6^a^	18.45±0.13^a^	33.68±0.26^a^
200	13.65±0.53^a^	44.60±3.35^a^	56.68±0.6^a^	18.83±0.19^a^	33.25±0.38^a^
500	14.85±0.51^a^	44.05±2.68^a^	52.28±0.6^a^	17.57±0.30^a^	33.65±0.18^a^
1000	15.23±0.31^a^	40.13±1.34^a^	55.60±0.7^a^	18.55±0.14^a^	33.35±0.31^a^
2000	14.37±0.30^a^	42.13±1.63^a^	54.80±3.4^a^	17.33±0.17^a^	32.80±1.25^a^
3000	14.88±0.31^a^	43.2±0.84^a^	52.28±0.4^a^	17.92±0.27^a^	34.33±0.42^a^
4000	13.85±0.39^a^	41.93 ±2.49^a^	54.10±0.6^a^	17.82±0.15^a^	33.00±0.23^a^
5000	14.80±0.67^a^	45.20 ±1.81^x^	53.33±1.0^a^	18.07±0.23^a^	33.77±0.22^a^

Values are haematological parameters and are expressed as Mean ± S.D. Values with the same superscript as Control are not significantly different. Values with superscript x are significantly increased when compared to control while values with superscript y are significantly decreased when compared to control. HGT = Haemoglobin concentration, HCT = Haematocrit (Packed cell volume), MCV = Mean Corpuscular Volume, MCH = Mean Corpuscular Haemoglobin, MCHC = Mean Corpuscular Haemoglobin Concentration

As compared to the control, Table 8a showed the lymphocytes count at doses of 5000 mg/kg body weight aqueous extract was 71.90 % and was significant increased compared with the control at 61.63 %. The monocytes count at doses of 4000 and 5000 mg/kg body weight were increased compared with the control; the monocytes count (16.15 %) was two times higher than the control (7.43 %) at dose 5000 mg/Kg. The granulocytes count was 18.95 % at dose 5000 mg/Kg and was significantly decreased compared with control (30.95 %). However, the graded doses of methanol extract were not significantly different from the control across all parameters **(**Table 8b**)**

**Table 8a:** Effect of aqueous extract of *Tetracera alniifolia* on platelets and white blood cells count.

DOSE mg/kg bw	WBC (X10^3^) cells/µL	LYM %	MONO %	GR %	PLT (X10^3^) cells/µL
CONTROL	11.60±2.08^a^	61.63±0.91^a^	7.43±1.24^a^	30.95±1.83^a^	483.67±218.63^a^
200	8.48 ±1.38^a^	60.00±5.59^a^	10.40±2.20^a^	29.60±1.44^a^	420.00±34.84^a^
500	13.75 ±0.98^a^	69.03±1.25^a^	8.40±0.80^a^	28.83±1.40^a^	453.00± 17.61^a^
1000	11.90±1.43^a^	62.18±4.71^a^	9.93±1.25^a^	27.32±5.33^a^	475.25 ±59.03^a^
2000	14.05 ±1.00^a^	65.45±5.08^a^	9.93±0.78^a^	24.63±2.03^a^	515.75 ±40.51^a^
3000	10.30± 1.30^a^	66.83±2.06^a^	10.93±1.48^a^	31.25±1.62^a^	409.00 ±14.75^a^
4000	11.25 ±0.31^a^	61.65±0.74^a^	12.05±1.50^x^	31.95±1.92^a^	543.00±33.74^a^
5000	17.45±3.12^a^	71.90±0.41^x^	16.15±1.52^x^	18.95±1.62 ^y^	514.00±40.01^a^

**Table 8b:** Effect of methanol extract of *Tetracera alnifolia* on platelet and white blood cells count.

DOSE mg/kg bw	WBC cells/µl	LYM %	MONO %	GR %	PLT cells/µl
CONTROL	11.60±2.08^a^	61.63±0.91^a^	7.43±1.24^a^	30.95±1.83^a^	483.67±218.63^a^
200	11. 98±1.10^a^	63.55±3.43^a^	9.10±2.45^a^	23.55±3.90^a^	526.00 ±43.11^a^
500	12.05±1.86^a^	70.75±2.29^a^	9.55±0.85^a^	29.70±1.84^a^	487.00 ±18.20^a^
1000	13.57±0.26^a^	67.60±3.09^a^	8.28±0.41^a^	24.13±2.80^a^	455.67±21.94^a^
2000	11.08 ±1.20^a^	60.43±4.72^a^	8.67±0.38^a^	30.90±4.51^a^	503.50±35.80^a^
3000	11.24 ±2.69^a^	61.28±3.53^a^	7.43±0.82^a^	31.31±3.13^a^	377.00 ±45.36^a^
4000	9.45 ±0.55^a^	62.50±3.67^a^	9.43±0.74^a^	28.33±3.41^a^	466.50 ±88.50^a^
5000	10.88±1.43^a^	64.40±2.72^a^	6.77±0.63^a^	28.83±3.30^a^	418.25±23.26^a^

Values are platelet, total and differential white blood cell count and are expressed as Mean ± S.D. Values with the same superscript as Control are not significantly different. Values with superscript x are significantly increased when compared to control while values with superscript y are significantly decreased when compared to control. WBC = White blood cell (total), LYM = lymphocyte, MONO = monocyte, GR = granulocyte, PLT = Platelet.

### 3.5 Effect of aqueous and methanol extract of *Tetracera alnifolia* on the histopathology of heart, liver and kidney in Albino rat

Histopathological evaluation of the effect of doses 200–5000 mg/Kg body weight aqueous and methanol root extracts of *Tetracera alnifolia* on the heart (Fig. 2a, Fig. 2b, Fig. 2c) revealed each extract induced coronary vascular dilation and congestion. The myocardium appeared normal. The methanol extract (Fig. 2c) had equal and safe effect as the aqueous extracts; however, it induced higher vascular changes than the aqueous extract. In the liver (Fig. 3a, Fig. 3b, Fig. 3c), the hepatocytes appeared normal with no instances of enlargement or reduction in the size of the hepatocytes. There was activation of immune cells (kupffer cells and lymphocytes); however, the aqueous extract (Fig. 3b) had higher activation strength in the liver than the methanol extract. (Fig. 3c). Administration of both extracts did not induce any toxic damage to the nephrons in the kidney as they appeared normal (Fig. 4a, Fig. 4b, Fig. 4c**)**.

**Fig. 1:**
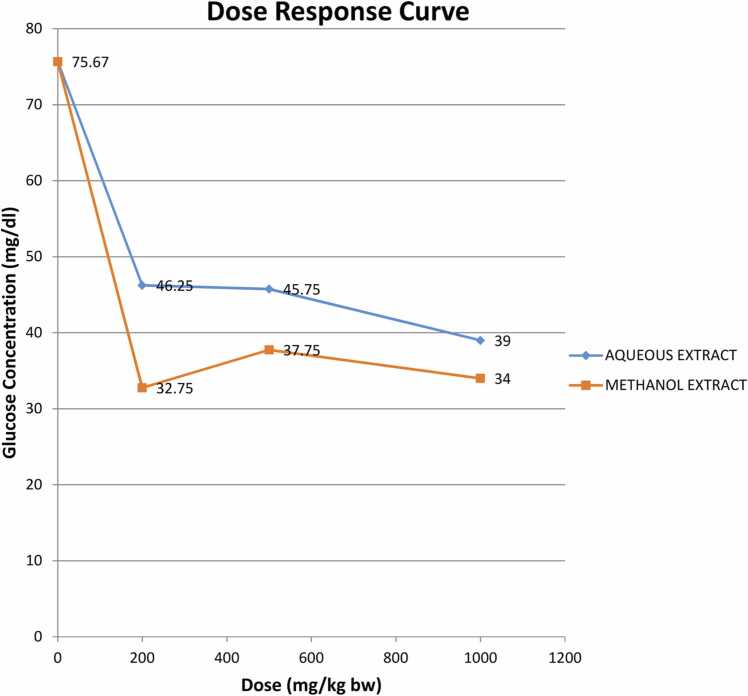
Dose response curve fast blood glucose concentration for various doses of aqueous and methanol extract.

**Fig. 2a:**
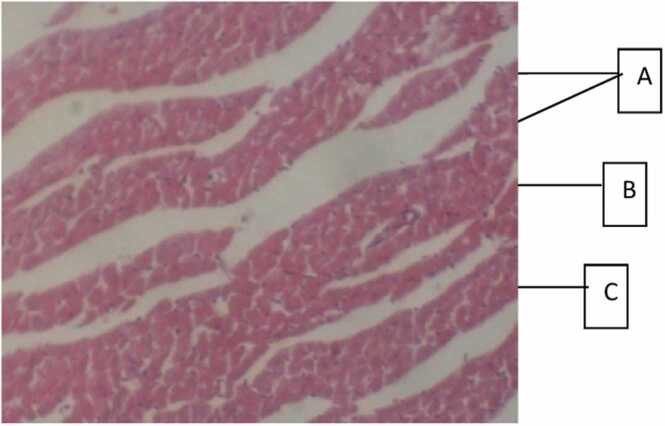
**Effect of aqueous and methanol extract of*****Tetracera alnifolia*****on the histopathology of heart in Albino rat.** Photomicrograph section of the heart (Control group): Heart composed of A, bundles of myocardial fibres, B, coronary vessel and C, interstitial spaces.

**Fig. 2b:**
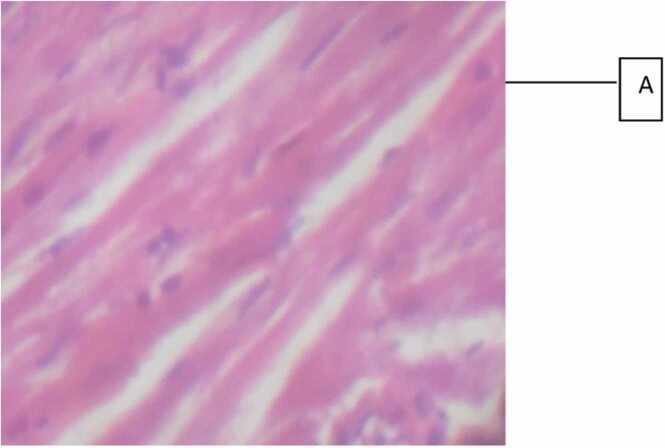
**Effect of aqueous and methanol extract of*****Tetracera alnifolia*****on the histopathology of heart in Albino rat.** Photomicrograph section of the heart (Aqueous extract) showing A, normal myocardial architecture,.

**Fig. 2c:**
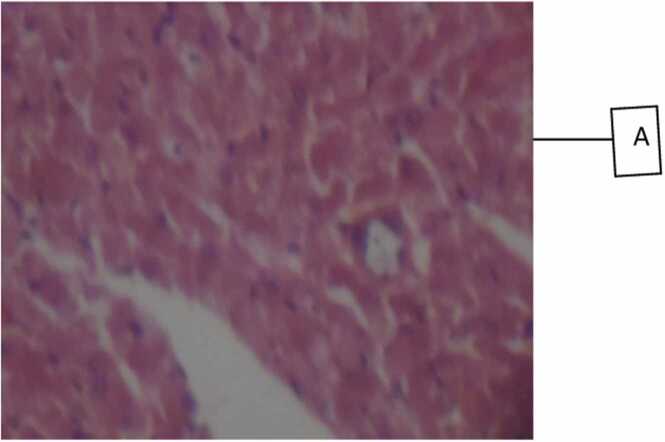
**Effect of aqueous and methanol extract of*****Tetracera alnifolia*****on the histopathology of heart in Albino rat.** Photomicrograph section of the heart (Methanol extract) showing A, normal myocardiac architecture.

**Fig. 3a:**
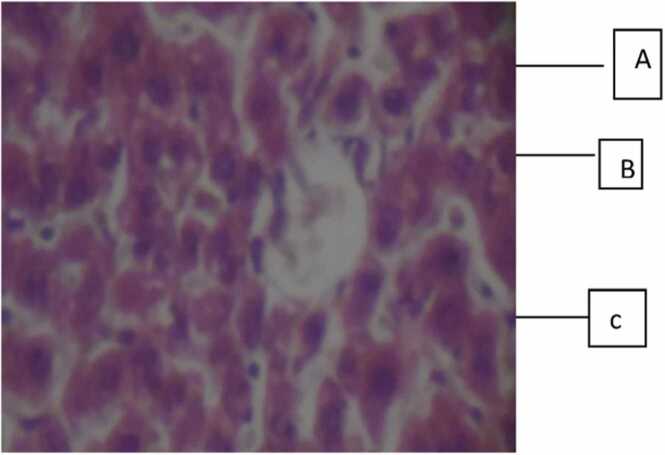
**Effect of aqueous and methanol extract of*****Tetracera alnifolia*****on the histopathology of liver in Albino rat.** Photomicrograph section of the liver (Control Group) showing central vein(A), Hepatocytes (B) and sinusoids (C).

**Fig. 3b:**
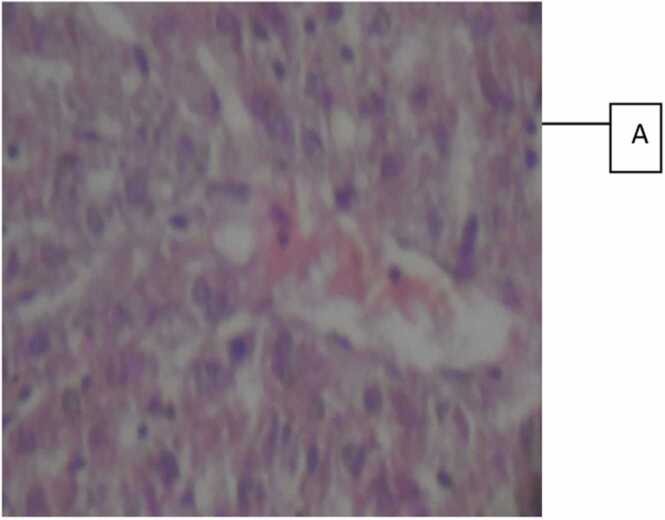
**Effect of aqueous and methanol extract of*****Tetracera alnifolia*****on the histopathology of liver in Albino rat.** Photomicrograph section of the liver (aqueous extract) showing A, mild portal congestion and mild kupffer cell.

**Fig. 3c:**
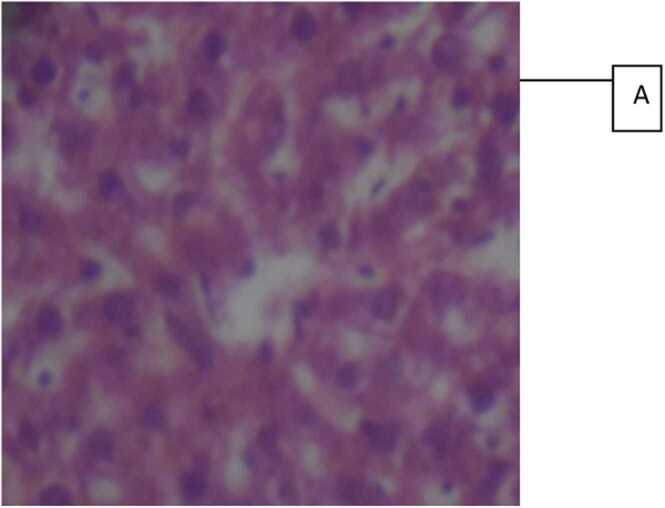
**Effect of aqueous and methanol extract of*****Tetracera alnifolia*****on the histopathology of liver in Albino rat.** Photomicrograph section of the liver (Methanol extract) showing A, normal hepatocyte architecture.

**Fig. 4a:**
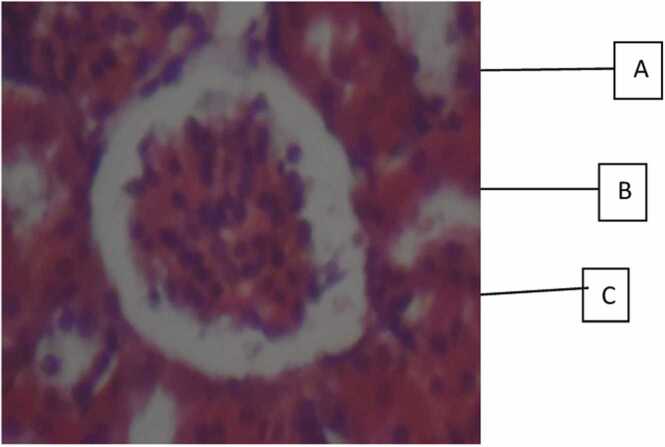
**Effect of aqueous and methanol extract of*****Tetracera alnifolia*****on the histopathology of kidney in Albino rat.** Photomicrograph section of the kidney (Control group) showing glomerulus A, tubules B and interstitial space C.

**Fig. 4b:**
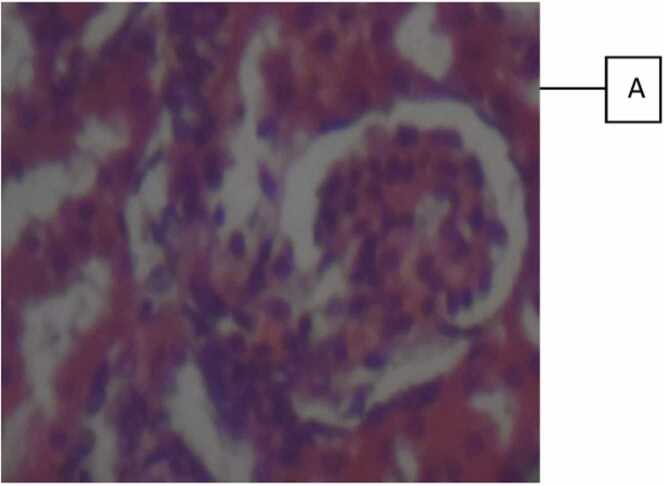
**Effect of aqueous and methanol extract of*****Tetracera alnifolia*****on the histopathology of kidney in Albino rat.** Photomicrograph section of the kidney (Aqueous extract) kidney showing A, normal renal architecture.

**Fig. 4c:**
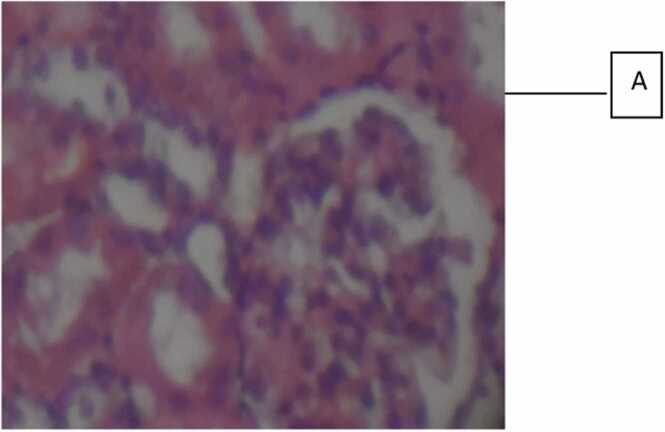
**Effect of aqueous and methanol extract of*****Tetracera alnifolia*****on the histopathology of kidney in Albino rat.** Photomicrograph section of the kidney (Methanol extract) showing A, mild interstitial congestion.

### 3.6 Dose-response study

The dose response curve (Fig. 1) showed a gradual decrease in fasting blood glucose concentration over the period of administration of each extract (15 days) in albino rats. At doses 200, 500, and 1000 mg/kg body weight, each extract reduced the blood glucose levels in the rats. However, the hypoglycemic effect of the methanol extract was better.

### 3.7 Biochemical profile analysis

There was no significant difference in the concentrations of cholesterol, triglycerides, HDL and LDL cholesterol, malondialdehyde, superoxide dismutase, reduced glutathione, and glutathione peroxidase activity following the administration of 200 mg/kg B. W. of each extract. However, the catalase activity for aqueous and methanol extract was 56.38U/mg protein and 66.14 U/mg protein compared with control at 30.25 U/mg protein indicating a significant increase in catalase activity and over two times increase for the methanol extract. (Table 9).

**Table 9:** Effect of aqueous and methanol extract of *Tetracera alnifolia* on biochemical parameters in Wistar albino rats.

Biochemical Parameter	Control	Aqueous extract 200 mg/kg bw	Methanol extract 200 mg/kg bw
Cholesterol (mg/dl)	36.40±7.82^a^	30.34± 4.74^a^	45.64±6.20^a^
Tryglyceride (mg/dl)	30.34±4.74^a^	32.26±12.42^a^	38.40±3.57^a^
HDL-Cholesterol (mg/dl)	12.46±0.37^a^	12.50±0.55^a^	12.88±0.69^a^
LDL-Cholesterol (mg/dl)	17.81±8.51^a^	24.77±4.31^a^	25.07±16.17^a^
Superoxide dismutase (Unit/mg Protein)	392.32±10.76^a^	393.67±15.53^a^	392.37±28.69^a^
Glutathione peroxidase (unit/mg protein)	281.56±7.23^a^	220.55±4.94^a^	299.47±3.61^a^
Reduced glutathione (g/l)	29.33±1.76^a^	34.60±4.70^a^	34.40±4.73^a^
Catalase (unit/mg protein)	30.25±5.72^a^	56.38±9.98^x^	66.14±2.76^x^
Malondialdehyde (moles/mg Protein)	1.95±1.05^a^	1.19±0.55^a^	1.77±0.18^a^

The values are biochemical parameter and are presented as Mean ± Standard Error of Mean. Values that have the same superscript as the control do not differ significantly. In comparison to the control, values with superscript x show a significant increase, while values with superscript y show a significant decrease

## 4 Discussion

The result of the proximate analysis of the pulverized root of *Tetracera alnifolia* revealed that carbohydrate was the highest nutrient while moisture content was the least **(**Table 1). The findings in this current study corroborates with the findings of [54] except for moisture, protein and crude fat. The local uses of the various part of this plant for treatment of diabetes, arthritis and anemia may be adduced to its nutritional composition.

Phytochemical screening of both extracts indicated the presence of saponins, tannins, alkaloids, flavonoids, phenols, cardiac glycosides. anthraquinones and steroids in both extracts (Table 2). [17] have reported the presence of saponin and cardiac glycosides in the stem bark of *Teracera alnifolia*, alkaloid and tannins were absent while anthraquinone was inconclusive. In this study, saponins, cardiac glycosides, tannins, alkaloids and anthraquinones were all present in the root of this plant. [15] reported the presence of alkaloids, saponins, tannins, anthraquinones, flavonoids, carotenoids and steroids in the leaves of *Tetrecera alnifolia*. In this study, these phytochemicals were present except for carotenoids. Furthermore, saponins, terpenoids, cardiac glycosides, polyphenols and flavonoids have been reported present in the stem bark of *Tetracera alnifolia* while alkaloids, tannins and anthraquinone were absent [55]. In this study all the phytochemicals were present in the root of *Tetracera alnofolia* except for terpenoids which was not investigated in this study.

Plants have medicinal value because of some phytochemicals that are present in their root, stem, leaves, fruits, and seeds, among other parts of the plant. These plant-based compounds have the ability to affect human physiological function. The most important of these bioactive compounds are alkaloids, flavonoids, tannins and phenolic compounds. [56] reported that the presence of saponins, polyphenols and cardiac glycosides may be responsible for the acclaimed anti-anaemic potential of plants used in traditional medicine. Alkaloids and flavonoids have been reported to be powerful antioxidants [5]. Hypoglycemic potential of plants has been associated with alkaloids, phenols, tannins, flavonoids and phlobotannins. [57]. Cardiac glycosides are known to increase contraction of heart muscle which improves cardiac output and reduces distension of the heart; hence they are used in the treatment of congestive heart failure and cardiac arrhythmia [58]. Saponins are used in hypercholestrolemia, hyperglycaemia, as antioxidant, anti-cancer, anti-inflammatory agent and for weight loss [59]. The presence of these phytochemicals in *Tetracera alnifolia* may therefore be responsible for its therapeutic use for the treatment and management of diseases such as anaemia, rheumatism, arthritis and diabetes in traditional medicine.

Acute toxicity study examines a chemical's ability to produce negative effects following oral or topical administration of a single or multiple dose within a 24-hour period or a 4-hour inhalation exposure (R). Acute toxicity gives clues in the range of doses that could be toxic to animals. Lethal dose (LD_50_) which refers to the quantity of a substance administered all at once that results in the death of half the number of a group of experimental animals, is one method of evaluating the acute toxicity (immediate poisoning potential) of a substance. From the result of the acute toxicity study, there was no mortality and also no other signs of toxicity for each extract at the different doses for up to 72 hours after administration (Table 3). Hence, the oral LD_50_ for aqueous and methanol root extracts of *Tetracera alnifolia* in albino rats was greater than 5000 mg/kg body weight.

Using the Hodge and Sterner toxicity scale [60], test drugs administered orally to rats are categorized as follows: less than 1 mg/kg is extremely toxic, 1–50 mg/kg is highly toxic, 50–500 mg/kg is moderately toxic, 500–5000 mg/kg is slightly toxic, 5000–15,000 mg/kg is practically nontoxic, and greater than 15,000 mg/kg is harmless. This implies the oral LD_50_ of *Tetracera alnifolia* root extracts in Wistar rats, which was greater than 5000 mg/kg body weight in this study, was practically non-toxic. The lethal dose of aqueous extract of the leaves of *Teracera alnifolia* in male and female rats has been report as greater than 2000mg/Kg body weight and regarded slightly toxic [61]. The oral LD_50_ (rats) of aqueous and 50 % ethanol stem bark of *Triplochiton scleraxylon K. Schum* and methanol extract of leaves of *Euphorbia hirta L* have been reported as greater than 5000 mg/kg [62].

Cardiac enzymes such as AST, CK, and LD are very important parameters in the diagnosis of cardiac diseases such as myocardial infarction. These enzymes are firmly attached to the cardiac muscle tissues' contractile apparatus, and any significant damage to the heart will cause the release of these enzymes into the plasma [63]. The effect of drug transformation and metabolism on the status of cardiac cells could also be assessed by evaluating aspartate aminotransferase, creatine kinase and lactate dehydrogenase activities in the plasma [64]. Serum creatine kinase is a more sensitive indicator in early stage of myocardial ischemia while peak rise in lactate dehydrogenase is roughly proportional to the extent of injury to the myocardial tissue [65]. With the exception of dose 5000 mg/kg body weight group, where it did not differ significantly from the control group, creatine kinase was significantly reduced in at doses 200–4000 mg/Kg aqueous extract with the least reduction at 200 mg/Kg. However, there was no significant difference observed for the methanol extract (Table 4a, Table 4b). This may imply that the aqueous extract of the root of *Tetracera alnifolia* has a greater potential of lowering the intrinsic factors that cause myocardial infarction. Also, it may suggest that both extracts did not produce any serious injury to muscle tissues of the heart that could provoke the release of creatine kinase into the plasma. Plasma lactate dehydrogenase activity of the various groups of experimental animals administered graded doses of each extract was not significantly different from the control. This may be attributed to the fact that the extracts did not provoke injury to the myocardium. Aspartate aminotransferase activity was only significantly increased at doses 4000 and 5000 mg/Kg for both aqueous extract and methanol extract (Table 4a, Table 4b). Increase in plasma aspartate aminotransferase activity may not be adduced to leakage from the myocardium since this enzyme is present in the cytoplasm and mitochondria of several organs including skeletal muscles, pancreas, heart, liver, kidney, skeletal and erythrocytes [66]. Furthermore, there was no concurrent significant increase in the other cardiac enzymes; lactate dehydrogenase or creatine kinase activity, indicating the increase in AST activity may result from the other organs.

The kidney is one of the organs responsible for the removal of waste products including urea and creatinine. It also regulates electrolytes concentrations in the body. Several factors can impair kidney function and can eventually lead to kidney failure. Kidney failure can be caused by medications, including phytochemicals that damage the kidney's tubules. Consequently, this can affect three important processes in the kidney: glomerular filtration, tubular reabsorption and secretion. Renal function tests are usually required to assess the normal functioning capacity of the nephron; functioning unit of the kidney [67]. Renal function gives an indication of the state of the kidney and its role in removing waste products, controlling the body’s fluid balance and regulating electrolytes balance [68].

Plasma creatinine is a reliable indicator of glomerular filtration rate (GFR). This is due to the fact that unless there is a change in GFR due to impaired renal function, the concentration of plasma creatinine remains relatively constant under normal conditions. According to [69], GFR and serum creatinine concentration are inversely proportional; a decrease in serum creatinine corresponds to an increase in GFR and vice versa. In this study, plasma concentration of creatinine for the graded doses for each extract were not significantly different and suggests a normal GFR **(**Table 5a, Table 5b). The main byproduct of the metabolism of nitrogen and amino acids is urea, which is produced in greater quantities than it is excreted in the urine. As a measure of GFR, plasma urea concentration is less reliable than creatinine because it diffuses back into renal tubular cells and is dependent on liver function, the amount of protein in the diet, and oxidation [70]. Nevertheless, when assessing renal function, the estimation of urea and creatinine complement each another. *Tetracera alnifolia* (root) graded doses of aqueous and methanol extract did not significantly alter the urea levels when compared to the control group. The insignificant difference in plasma urea concentration obtained in this research study may suggest that both extracts had no effect on the metabolism and excretion of urea

Maintaining osmotic balance and regulation of ionic charges determine the concentration of electrolytes in blood. This balance is brought about by the kidney, lungs, and endocrine system working together [71]. The main cation in extracellular body fluid is sodium ion. It guards against excessive fluid loss and maintains the proper acid-base balance in the body. The glomerular filtration rate (GFR) and sodium ion concentration have a linear relationship; more sodium ion is excreted in urine when the GFR increases, and vice versa [72]. In this current study, both extracts had an insignificant difference on sodium ion concentration for the graded doses across each group (Table 5a, Table 5b**)**. This may suggest that both extracts did not compromise the body’s use and excretion of sodium ion and also renal function was not affected.

The primary intracellular cation that regulates intracellular osmotic pressure is potassium. It also plays a vital role in propagation of nerve impulses along the nerve and transmission to receptor cells [73]. Renal failure, improper use of drugs such as potassium ion sparing diuretics, hyperglycemia caused by insulin deficiency, Addison's disease, hypoaldosteronism, and excessive death of tissue may lead to hyperkalemia [74]. Numerous factors, including diuresis, diarrhea, absorption disorder syndrome, sweating and pyrexia, prolonged stress, poor eating habits, Cushing's syndrome, hyperaldosteronism, hepatic disease, use of drugs and steroid hormone, can result in hypokaelemia. In this investigation, the administration of the aqueous extract at a dose of 5000 mg/kg body weight resulted in a significant increase in plasma potassium, whereas the methanol extract was insignificant. (Table 5a, Table 5b). It implies doses below 5000 mg/kg body weight for the aqueous extract may be safer.

The main anion in extracellular fluid is chloride. It is essential for water balance, regulation of osmotic pressure and acid-base balance. Except in metabolic acidosis and alkalosis, abnormalities in chloride metabolism commonly exist alongside with abnormalities in sodium metabolism [75]. The administration of both extracts produced an insignificant difference in plasma chloride concentration across the various groups administered graded doses when compared to the control group (Table 5a, Table 5b). This insignificant difference in plasma chloride concentration by both extracts may suggest no abnormalities in chloride metabolism and excretion, and also a normal renal function.

The concentration of base left over after all acids stronger than carbonic acid have been neutralized is designated as the concentration of bicarbonate in plasma. It is known as the "alkali reserve" and refers to the reserve of alkali that is kept aside for the purpose of neutralizing strong acids. It plays a crucial role in maintaining the acid-base balance as part of the pH buffering system. The level of plasma bicarbonate is decreased in metabolic alkalosis and respiratory acidosis, but elevated in respiratory alkalosis and metabolic acidosis. In this study, administration of aqueous and methanol extract of root of *Tetracera alnifolia* had no significant difference on the various group of the experimental animals. This may suggest that the extract had no significant effect on the metabolism and excretion of bicarbonate ion.

The liver is essential for metabolism, digestion, detoxification, and the excretion of substances from the body. It's also a vital organ in maintenance, performing and regulation of the body's homeostasis [76]. The liver is subjected to long-term exposure to toxic substances, which can negatively affect its function by causing cell derangement, or the loss of functional cells [77]. Severe hepatic injury may be linked to a notable distortion of its functions due to the metabolism of some toxic bioactive compound in plants used as crude medicine and the liver's inability to eliminate the metabolic products [78]. The primary organ for protein synthesis is the liver. Decreased in the concentration of total protein in plasma can be caused by liver diseases, hypogammaglobulinemia, increased loss of protein from renal tubule, gastrointestinal and skin disorders, over hydration and poor synthesis of protein from malnutrition and malabsorption [79]. Increased concentration of plasma total protein can be caused by dehydration and increased immunoglobulin due to infection [80]. In this study, the aqueous and methanol extracts significantly reduced plasma protein at 5000 mg/kg body weight (Table 6a, Table 6b). However, the measurement of total protein in plasma alone may not be reliable indicator of an individual's metabolic state because the quantities of the different proteins do not increase or decrease in tandem with one another [79]. Plasma albumin level, the most abundant plasma protein, can provide more insight into the liver's synthetic capacity. It acts as a protein reserve, facilitates the transport of both endogenous and exogenous substances, and helps to maintain osmotic pressure [81]. If the liver's ability to synthesize albumin is compromised, the amount of albumin the liver produces is decreased. Both liver cirrhosis and hepatitis cause a decrease in serum albumin [82]. In this study, administration of aqueous and methanol extract had no significant difference in plasma albumin concentration except a slight reduction at dose 5000 mg/kg. This reduction may be adduced to reduced albumin synthesis and invariably reduced synthetic function of the liver. It may also imply that reduced plasma total protein by both extracts observed earlier may be due to reduced albumin concentration.

Bilirubin, a product of heme breakdown, is a helpful tool for assessing haemolytic anaemia and evaluating liver's excretory function. Elevated serum bilirubin indicates an increased production of bilirubin due to an increase breakdown of red blood cells or the ability of the liver to remove bilirubin has been compromised due to blocked bile duct or an underlining liver problem [83]. In this study, both extracts had an insignificant difference on the concentration of total and conjugated (direct) bilirubin for the graded doses (Table 6a, Table 6b). The insignificant difference in the concentrations of both total and direct bilirubin may suggest that both extracts had no effect on the metabolism and excretion of bilirubin. This may further suggest that there was no hepatobiliary obstruction in the liver and the excretory function of the liver was not compromised.

The cytoplasm and mitochondrium of cells of the liver, heart, skeletal muscles, kidney, brain, lungs, pancreas, white blood cells, and red blood cells contain the enzyme aspartate aminotransferase. Alanine aminotransferase is found in the cytosol of hepatocytes and it is a more sensitive marker of hepatocellular damage than AST. Aspartate aminotransferase, alanine aminotransferase, alkaline phosphatase and L-γ-glutamyltransferase are the serum enzymes present normally in the liver in high concentration but low in the serum. When necrosis occurs or hepatic cells’ plasma membrane are damage, these enzymes normally located in the cytosol leak into the blood stream; raising plasma concentration of these enzymes. Their estimation in the plasm is a useful quantitative marker of the extent and type of hepatocellular damage [84]. In this study, administration of aqueous and methanol extract significantly increased aspartate aminotransferase activity at doses 4000and 5000 mg/kg body weight compared to control group as earlier stated. Administration of each extract significantly increase ALT activity at dose 5000 mg/kg body weight. This increase observed in these liver enzymes activity particularly ALT may not necessarily connote liver disease because slight increase in AST and ALT activities within 1.5 times the upper limits of normal do not necessarily indicate liver disease [85]. Also, other non-hepatic disorders may influence liver enzymes activities in plasma as a result of the presence of these enzymes in other tissues of the body. Alkaline phosphatase is a marker enzyme for the plasma membrane and endoplasmic reticulum [86]. It is used to assess plasma membrane integrity of the liver. In numerous medical conditions, particularly in liver and bone disorders, its activity is elevated. It is a helpful diagnostic tool for osteoblastic bone disease and cholestatic hepatobiliary lesions assessment and follow-up. Administration of each extract only resulted to a significant increase in ALP activity compared to the control for at doses 5000 mg/kg (Table 6a, Table 6b). This implies that at high dosage both extracts may lead to either a bone or liver disease. In this study, administration of aqueous and methanol extracts had an insignificant difference when compared to the control for GGT activity for the various doses.

Blood serves as a good pathological insight into how the body functions. Blood's cellular components are useful in immunotoxicology to assess a compound's potential for immunotoxicity [87]. Haematological parameters are helpful indicators that can be used to evaluate a plant extract's potential for toxicity in living systems as well as to clarify how a chemical compound or plant extract is associated with blood cell component and function [88]. *Tetracera alnifolia* methanol extract slightly increased the haematocrit (HCT) value at a dose of 5000 mg/kg body weight (Table 7a, Table 7b). The insignificant difference in haematocrit (HCT) values, haemoglobin concentration (HGT), mean corpuscular volume (MCV), mean corpuscular haemoglobin (MCH) and mean corpuscular haemoglobin concentration (MCHC) may suggest that both extracts did not compromise the process of erythropoiesis and may not cause haemolytic anaemia.

Leucocytes are the first line of cellular defense that respond to infectious agent, tissue injury or inflammatory process [89]. In this study, administration of methanol extracts did not significantly increase or decrease the total white blood cells, lymphocyte, monocytes and granulocytes count. Aqueous extract significantly increased the lymphocytes count at 5000 mg/Kg and monocytes count at doses 4000 mg/Kg and 5000 mg/kg body weight while granulocytes was decreased at 5000 mg/kg body weight when compared to the control (Table 8a, Table 8b). Platelets are cell fragments that play a vital role in blood clotting. They also initiate the repair of blood vessels and help in maintaining normal homeostasis. The insignificant difference in platelet count for the various doses of aqueous and methanol extract compared to the control indicates each extract may not adversely affect the production of platelets.

Dose response study revealed both extracts reduced blood glucose at doses 200, 500 and 1000 mg/kg body weight (Fig. 1). However, for convenience 200 mg/kg body weight was used for the biochemical study. Hypoglycemic potential of plants has been associated with some phytochemicals including flavonoids, phenols, alkaloids, tannins and phlobatannins [90]. The hypoglycemic effect of both extracts may be adduced to the presence of these phytochemicals in both extracts. *Teracera alnifolia* have been reported to have hypoglycemic effect [17].

Cardiovascular disease is characterized by high levels of plasma total cholesterol, triglycerides and LDL- cholesterol [91]. In this study, administration of 200 mg/kg body weight of both extracts did not significantly increase or decrease the concentration cholesterol, triglycerides, LDL-cholesterol and HDL-cholesterol when compared with the control (Table 9). This insignificant difference may suggest that both extracts had no effect in the metabolism and utilization of these lipids and lipoproteins and also suggest less risk of developing cardiovascular diseases in the experimental animals.

Oxidative stress occurs as a result of an excessive production of reactive oxygen species above the body’s antioxidant capacity and has been implicated in the development of many pathophysiological conditions including diabetes, hypertension, atherosclerosis, and cancer [92]. The formation of reactive oxygen species is preserved by an antioxidant system that includes non-enzymatic antioxidant (ascorbic acid, glutathione, and tocopherols), enzymes regenerating the reduced forms of antioxidant and the reactive oxygen species (ROS) scavenging enzymes such as superoxide dismutase, glutathione peroxidase and catalase. Superoxide dismutase converts superoxide anion to hydrogen peroxide in order to scavenge reactive oxygen species [93]. Decrease in superoxide dismutase activity may lead to accumulation of ROS which may result in oxidative stress. Superoxide dismutase activity for each extracts were not significant different from the control (Table 9). Glutathione peroxidase and glutathione-S-transferase (GST) work together with glutathione in the decomposition of hydrogen peroxide or other organic hydroperoxides to non-toxic products at the expense of the reduced glutathione [94]. In this study, the aqueous and methanol extract did not significantly increase or decrease reduced glutathione concentration and glutathione peroxidase activity. This insignificant difference may suggest that both extracts did not compromise the metabolism of glutathione and its antioxidant function was not affected. Catalase is a heme protein and it scavenges the hydrogen peroxide radicals that are formed during various metabolic reactions in the cells [95]. Catalase is the second enzyme involved in the antioxidant defenses by converting hydrogen peroxide to water and oxygen. In this study, administration of both extracts significantly increased plasma catalase activity. This increase may suggest that each extract confer a protective antioxidant activity against reactive oxygen species (ROS) in the experimental animals. Malondialdehyde is a stable metabolite of free radical-mediated lipid peroxidation cascade [96]. Hence, it is a major marker of lipid peroxidation. In this study, administration of the extracts resulted in an insignificant difference when compared to the control group. This is an indication that the extracts did not alter lipid peroxidation statues in the experimental rats.

This research study showed that on short term exposure (acute toxicity), aqueous and methanol extracts of root of *Tetracera alnifolia* were not toxic and well tolerated in Wistar albino rats. Sub-acute toxicity study for twenty-eight days revealed both extracts were well tolerated and not toxic to the liver except at doses 5000 mg/kg body weight where slight toxicity is suspected due to increase in plasma AST, ALT and ALP accompanied with a decrease in total protein and albumin concentration. Concomitant increase in AST, ALT and ALP values are implicated in liver disease. The increase in these enzymes may indicate leakage from the liver. However, histopathological study of liver tissue (Fig. 3a, Fig. 3b, Fig. 3c) showed no signs of hepatocytes damage, hepatomegaly, steatosis (microvesticular and macrovesticular) and cholestasis.

Administration of aqueous and methanol extracts during this period of sub- acute study were not toxic to the kidney. Sodium ion, bicarbonate ion, chloride ion, urea and creatinine plasma concentration were generally not significantly different from the control group. Histopathological study of kidney tissue (Fig. 4a, Fig. 4b, Fig. 4c) showed normal nephron with no signs of kidney damage, toxic nephrosis and nephritis [97]. Aqueous and methanol extracts administration did not increase the heart enzymes lactate dehydrogenase and creatine kinase that were evaluated in this study. Reduction in creatine kinase by the aqueous extract may suggest protection of the myocardium and reduce the risk of myocardial infarction. Histological study revealed normal cardiomyocytes and that each extract induced increase vasoactive effect of dilation and congestion which increase blood flow (Fig. 2a, Fig. 2b, Fig. 2c) and there were no signs of pathology: cardiomyopathy, cardiomegaly, inflammatory cellular infiltration, fibrosis and necrosis [98]. This may be attributed to the presence of cardiac glycosides in both extracts as cardiac glycosides are known to increase contraction of heart muscles which improves cardiac output and reduces distention of the heart muscles. Hematological assessment using haematological parameters showed that both extracts were not toxic to the blood. Both extracts did not compromise haematopoiesis and the various functions of the blood cells. Only slight variations of some haematological parameter were observed particularly for the aqueous extract at 5000 mg/Kg B.W. Evaluation of some biochemical parameters using dose 200 mg/kg body weight for each extract suggests the experimental animals seem safe from the risk of developing cardiovascular disease resulting from derailment in lipid metabolism and utilization and may possess antioxidant property.

## 5 Conclusion

In conclusion, short term and mid-term administration of aqueous and methanol extracts of the root of *Tetracera alnifolia* were generally not toxic to the heart, liver, kidney and blood of male albino rats. However, both extracts appeared slightly toxic to the liver at dose 5000 mg/kg body weight. In addition, analysis of biochemical parameter revealed that both extracts had no effect on lipid peroxidation and showed possible antioxidant property. The result from this research may help validate the safety of the oral use of this plant in traditional medicine. Further research should be done to evaluate the possibility of sub- chronic and chronic toxicity in experimental animals and also a more detailed study of its anti-glycemic property in the treatment of diabetes mellitus as well as other ailment mentioned in traditional medicine.

## Funding

This research did not receive any specific grant from funding agencies in the public, commercial, or not-for-profit sectors

## CRediT authorship contribution statement

**Ufuoma Augustina Igbuku:** Writing – review & editing, Writing – original draft, Visualization, Validation. **Endurance Fegor Isoje:** Writing – review & editing, Writing – original draft, Visualization, Validation. **Joseph Oghenewogaga Owheruo:** Writing – review & editing, Writing – original draft, Visualization, Validation. **Patrick Othuke Akpoghelie:** Writing – review & editing, Writing – original draft, Visualization, Validation. **Irene Ebosereme Ainyanbhor:** Writing – review & editing, Writing – original draft, Visualization, Validation, Software, Resources, Project administration, Methodology, Investigation, Formal analysis, Data curation, Conceptualization. **Huzaifa Umar:** Writing – review & editing, Writing – original draft, Visualization, Validation. **Arthur Efeoghene Athan Essaghah:** Writing – review & editing, Writing – original draft, Visualization, Validation. **Emad Yousif:** Writing – review & editing, Writing – original draft, Visualization, Validation. **Great Iruoghene Edo:** Writing – review & editing, Writing – original draft, Visualization, Validation, Supervision, Software, Resources, Project administration, Methodology, Investigation, Funding acquisition, Formal analysis, Data curation, Conceptualization. **Iyere Osolase Onoagbe:** Writing – review & editing, Writing – original draft, Visualization, Validation, Supervision, Resources, Project administration, Methodology, Formal analysis, Data curation, Conceptualization.

## Declaration of Competing Interest

The authors declare that they have no known competing financial interests or personal relationships that could have appeared to influence the work reported in this paper.

## Data Availability

The authors do not have permission to share data.
